# Difference in physiological responses of growth, photosynthesis and calcification of the coccolithophore *Emiliania huxleyi* to acidification by acid and CO_2_ enrichment

**DOI:** 10.1007/s11120-014-9976-9

**Published:** 2014-02-06

**Authors:** Shin-ya Fukuda, Yurina Suzuki, Yoshihiro Shiraiwa

**Affiliations:** 1 Faculty of Life and Environmental Sciences, University of Tsukuba, 1-1-1 Tennodai, Tsukuba, Japan; 2 CREST, JST, 1-1-1 Tennodai, Tsukuba, Japan

**Keywords:** Acidification, CO_2_ enrichment, Calcification, *Emiliania huxleyi*, pH effect, Photosynthesis

## Abstract

Ocean acidification, one of the great global environmental issues at present, is expected to result in serious damage on marine calcareous organisms such as corals and calcifying algae, which potentially release huge amounts of CO_2_ from the ocean to the atmosphere. The coccolithophore, *Emiliania huxleyi* (Haptophyceae), which frequently produces blooms, has greatly contributed to the biological CO_2_ pump. This study was aimed at analyzing effects of how *E. huxleyi* responds to acidification. Acidification was performed by two methods, namely by just adding HCl under bubbling ordinary air at 8.2–8.4, 7.6–7.8 and 7.1–7.3 (acidification by HCl) and by bubbling with ordinary air or with increased CO_2_ concentration such as 406, 816 and 1,192 ppm that maintained pH of the medium at 8.0–8.3, 7.6–7.9 and 7.5–7.7 (acidification by CO_2_ enrichment). As a result, cell growth and cellular calcification of *E. huxleyi* were strongly damaged by acidification by HCl, but not by acidification by CO_2_ enrichment. The activities of photosystems such as *F*_v_/*F*_m_ and ϕPSII were not affected by any acidification conditions while photosynthetic O_2_ evolution was slightly stimulated. A ^45^Ca-radiotracer experiment revealed that Ca^2+^-uptake was strongly suppressed by acidification with HCl. This suppression recovered after increasing the dissolved inorganic carbon (DIC) concentration and further stimulated by an additional increase in DIC concentration. The production of storage and coccolith polysaccharides was increased by acidification by HCl and also highly stimulated by acidification with CO_2_ enrichment. The present study clearly showed that the coccolithophore, *E. huxleyi*, has an ability to respond positively to acidification with CO_2_ enrichment, but not just acidification.

## Introduction

The increase in atmospheric CO_2_ concentration is now recognized to have increased ocean acidification (Orr et al. 2005; Zeebe et al. 2008). Oceanic pH has already decreased 0.1 U ever since the industrial revolution in the eighteenth century, and it is speculated to decrease 0.5 U further by the end of the twenty-first century according to IPCC scenario. The pH of the surface ocean is estimated to decrease by 0.3–0.5 and 0.7–0.77 U relative to the present level by 2,100 (pH 7.6–7.9) and 2,300 (pH 7.33–7.5), respectively (Caldeira and Wickett 2003; Ross et al. 2011). Such rapid ocean acidification is believed to have negative influences on marine organism with calcifying organisms as prime targets for strong damage by acidification (Feely et al. 2004), e.g., the bleaching and reduction of coral reefs (Gattuso et al. 1998; Kleypas et al. 1999; Hoegh-Guldberg et al. 2007; Anthony et al. 2008; Kuffner et al. 2008; Veron et al. 2009). In addition, the shell of gastropod, *Littorina littorea*, and foraminifera are shown to lose hardness by acidification (Bibby et al. 2007; Bijma et al. 2002). The fertilization rate of sea urchin, *Psammechinus miliaris*, declined with acidification (Miles et al. 2007). Such influence of oceanic acidification is expected to affect the entire ecosystem and damage the oceanic environment. However, even under such circumstances, actual events caused by acidification have not been investigated thoroughly in individual organisms (Richier et al. 2010).

In particular, a marine calcifying haptophycean alga, *Emiliania huxleyi*, is affected by ocean acidification (Iglesias-Rodriguez et al. 2008; Langer et al. 2006; Riebesell et al. 2000) because *E. huxleyi* forms cell-covering, calcium carbonate crystals, called coccoliths. The alga is known to distribute widely in the world ocean, fix a large amount of carbon, produce a huge biomass and carry carbon from sea surface to the sediment by the biological CO_2_ pump (Liu et al. 2009). Therefore, *E. huxleyi* can be said to have played very important roles in the global carbon cycle. Riebesell et al. (2000) reported a reduction in calcification by *E. huxleyi* under future scenarios on ocean acidification. However, Iglesias-Rodriguez et al. (2008) observed enhanced calcification under elevated pCO_2_ in *E. huxleyi*. Hoppe et al. (2011) reported that *E. huxleyi* shows identical responses to elevated pCO_2_ in total alkalinity (TA) and dissolved inorganic carbon (DIC) manipulations. They also showed that different experimental protocols (e.g., continuous bubbling versus pre-bubbled) can lead to change in growth rates and other ecophysiological parameters.

The coccolithophore *E. huxleyi* has influenced the global climate for over 200 million years and therefore is thought to have played critical roles in the global carbon cycle. Even in the present ocean, the algae are widely distributed globally and it is well known that they fix a large amount of carbon, produce a huge biomass and carry carbon from the sea surface to the sediment by the biological CO_2_ pump (Liu et al. 2009). Recently, Read et al. (2013) reported the first haptophyte reference genome, from *E. huxleyi* CCMP1516, and sequences from 13 additional isolates. It revealed that a pan genome (core genes plus genes distributed variably between strains) is probably supported by an atypical compliment of respective sequences in the genome. They assumed that such a wide variation of genomes in *E. huxleyi* seems to be the reason for forming large-scale episodic blooms under a wide variety of environmental conditions.

In this study, we investigated the physiological response of the coccolithophore *E. huxleyi* to acidification by experimental acid enrichment (acidification by HCl) and by ventilation of air with elevated concentration of CO_2_ (acidification by CO_2_ enrichment). These conditions are not exactly the same as the ocean acidification conditions being observed in the ocean, but will give important information on how *E. huxleyi* will respond to acidification. Finally, we clearly show that just acidification caused by HCl is disadvantageous to *E. huxleyi*, but acidification by CO_2_ enrichment induced positive influence on photosynthesis and calcification of the organism. This study also proved clearly that the suppression of intracellular calcification by acidification in the coccolithophore is due to the reduction of HCO_3_^−^ supply, which is the substrate for intracellular calcium carbonate crystal production, because the suppression of calcification recovered following additional supply of bicarbonate ions.

## Materials and methods

### Material and culture conditions

The strain (NIES 837) of the coccolithophore *E. huxleyi* (Lohmann) Hay and Mohler (Haptophyta) used in this study was collected by Dr. I. Inouye in the South Pacific Ocean in 1990 and has been maintained at 20 °C under 16-h light/8-h dark regime in our laboratory. Cells were maintained in natural seawater for stock culture. For experimental culture, the medium used was an artificial seawater (Marine Art SF-1; produced by Tomita Seiyaku Co., Ltd., Tokushima, Japan, distributed by Osaka Yakken Co., Ltd., Osaka, Japan) enriched with a micronutrient mixture of the Erd-Schreiber’s medium (ESM) in which soil extracts are replaced with 10 nM sodium selenite according to Danbara and Shiraiwa (1999). ESM enrichment contains 28.7 μM (final concentration in the medium) K_2_HPO_4_, but not in the Marine Art SF. In all acidification experiments, cells were grown in the artificial seawater containing EMS medium (MA/ESM medium) under constant illumination at 100 μmol photons m^−2^ s^−1^ and 20 °C (standard condition). To avoid large changes in the pH of the medium during culture, both HEPES and Tris-buffer (final concentration, 10 mM each) were added to the medium by considering those buffers’ buffering ability and pKa values.

### Bubbling cultures with air and air containing elevated concentration of CO_2_

**Fig. 1:**
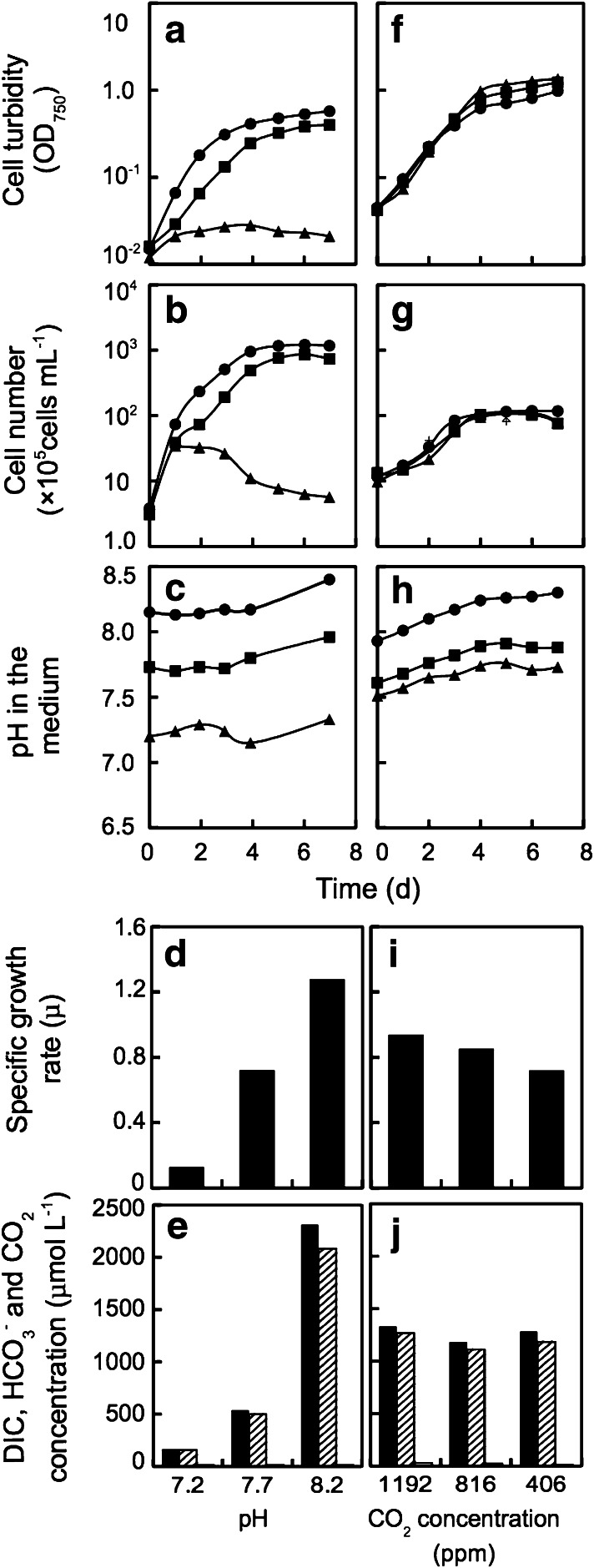
Effect of the acidification by HCl (**a**–**e**) and the ocean acidification conditions by elevating pCO_2_ (**f**–**j**) on the cell growth of the coccolithophore *E. huxleyi*. Before experiments, all cells had been grown at pH 8.2 under the bubbling of air containing 400 ppm CO_2_. Temperature was 20 °C. **a**, **f**, Change in turbidity; **b**, **g** change in cell number; **c**, **h** H in the medium. Initial pHs were set at 8.2 in **a** (*closed circles*), 7.7 in closed squares and 7.2 in closed triangles by HCl (**a**–**c**) and at 7.9 in *closed circles*, 7.6 in *closed squares* and 7.5 *closed triangles* by elevating pCO_2_ (**f**–**h**). **d**, **i** Specific growth rates (*μ*) calculated on the basis of cell number; **e**, **j** inorganic carbon concentrations in the medium at each pH and the elevated pCO_2_ concentration at 1 day. CO_2_ concentration was set at 15 μmol L^−1^ in all the conditions (*right column*). *Solid* (*left*) and stripe (*middle*) columns indicate total DIC and HCO_3_^−^ concentrations, respectively. DIC, bicarbonate and CO_2_ concentrations were calculated by a kind help of Dr. Midorikawa according to Leuker et al. (2000)

Tanks containing air with elevated concentrations of CO_2_, namely 406, 816 and 1192 ppm, were purchased from the company, Suzuki Shokan Ltd., Tsukuba, Japan. First, those gasses were bubbled through MA/EMS medium containing HEPES- and Tris-buffers (10 mM each) for 15 h as pre-bubbling for attaining equilibrium of CO_2_ between the bubbled gasses and the medium. The concentrations of respective DIC species in the medium shown in Fig. 1 and 6 were calculated according to Leuker et al. (2000) and CO_2_SYS, respectively. On the other hand, algal cells were grown separately with air in the MA/ESM medium under constant illumination at 100 μmol m^−2^ s^−1^ and 20 °C for 3 days. And then, an aliquot of the algal suspension was transferred to the previously prepared medium of which pH and *p*CO_2_ were already set by adding HCl or bubbling of air containing elevated CO_2_, as described above.

### Determination of the specific growth rate and microscopic observation

Cell turbidity of the culture was determined by measuring OD_750_ using a spectrophotometer (UV-1700, Shimadzu, Kyoto, Japan). Cell number was determined under a microscope by counting cells on a ready-made glass slide using a microscopic camera system and a counter. The specific growth rate (*μ*) was calculated by the equation of $\mu = { \ln }\left[ {{{\left( {m_{{t_{1} }} - m_{{t_{ 1} }} } \right)} \mathord{\left/ {\vphantom {{\left( {m_{{t_{1} }} - m_{{t_{ 1} }} } \right)} {(t_{ 2} - t_{ 1} )}}} \right. \kern-0pt} {(t_{ 2} - t_{ 1} )}}} \right],$ where *m*_x_ represents cell number at arbitrary time *t*_1_ and *t*_2_ (*t*_2_ > *t*_1_) during the logarithmic growth phase. Coccoliths covering cells were visualized under polarized light by a microscope (Olympus Ltd., Tokyo, Japan) equipped with a fluorescence microscope digital camera (Keyence, Osaka, Japan).

### Determination of photosynthetic activity

The algal cells were harvested from the culture and then centrifuged (700×*g* for 10 min at 15 °C) to obtain a cell pellet. After suspending cells in adequate buffers, photosynthetic O_2_ evolution activity was determined by a Clark-type oxygen electrode (Rank Brothers Co., Ltd., UK). The light intensity and temperature were maintained at 270 μ mol photons m^−2^ s^−1^ and 25 °C, respectively. The light source was a white LED lamp (Model HLV-24SW-3W, CCS, Kyoto, Japan).

### Determination of photosystem activity expressed with chlorophyll fluorescence parameters

Photosystems of *E. huxleyi* were characterized by the chlorophyll fluorescence method. First, chlorophyll concentration of cells was determined in 90 % methanol extracts by a spectrophotometer (UV-1700, Shimadzu, Kyoto, Japan) according to published procedures (Jeffrey 1972). Then algal concentration was adjusted to 5.0 μg Chl mL^−1^ in the MA/ESM medium (final phosphate concentration, 28.7 μM) at different pHs (7.2–8.2) for measurements. Photosystem activity was determined using a FluorCam (MF 701, Photon Systems Instruments, Bruno, Czech Republic), and the parameters of *F*_v_/*F*_m_ and ϕPSII were calculated by manufactured software attached to the apparatus. The duration and intensity of excitation light were 20 min and 100 μmol photons m^−2^ s^−1^, respectively, and of measured saturated pulsed light were 800 ms and 2,000 μmol photons m^−2^ s^−1^, respectively. Dissolved inorganic carbon (DIC) concentration was 2 mM, which was equilibrated with atmospheric CO_2_ concentration at pH 8.2.

### ^45^Ca uptake assay

Effect of pH on calcification was tested by a radiotracer method. The cells were harvested by centrifugation (700×*g* for 10 min at 15 °C) and re-suspended into the fresh experimental culture medium. The pH of the medium was adjusted at either pH 7.2, 7.7 or 8.2 by adding an aliquot of 0.2 N HCl. An aliquot of ^45^CaCl_2_ solution (Perkin-Elmer, Inc., Waltham, MA, USA) was directly injected into algal cell culture. Final concentration and the specific radioactivity of ^45^Ca in the medium were 10 mM and 20 MBq mmol^−1^, respectively. The algal suspension was continuously bubbled with ordinary air at a speed of 100 mL min^−1^. Subsequent experimental procedure for the determination of ^45^Ca uptake activity was according to the method of Kayano and Shiraiwa (2009). According to our previous results in the same strain of *E. huxleyi*, more than 95 % of calcium absorbed by cells is utilized for calcification **(**Satoh et al. 2009) and therefore the measurement of ^45^Ca-uptake could be used as a good parameter for calcification activity in this study.

### Assays

As the coccolith contains the coccolith polysaccharides, which are acid polysaccharides composed of uronic acids (Kayano and Shiraiwa 2009), uronic acid content was used as a parameter of acid polysaccharide (AP) production. The carbazole–H_2_SO_4_ assay (Bitter and Muir 1962) was used for the determination of uronic acid content using 0–90 μg mL^−1^ glucuronic acid (Chugai Pharmaceutical Co., Ltd., Tokyo, Japan) as a standard for calibration.

The amount of total polysaccharides (TP) included both AP and neutral polysaccharides (NP) composed of reducing sugars. TP was estimated as total sugars using a phenol–H_2_SO_4_ assay using 0–90 μg mL^−1^ glucose as a standard for calibration (Hodge and Hofreiter 1962). Then, the amount of NP was calculated by TP − AP.

The polysaccharides were analyzed by SDS-PAGE on a 15 % acrylamide gel. After electrophoresis, the gels were stained with Stains-all (Applichem GmbH, A1400.0001, Cheshire, USA) and Alcian blue (Sigma-Aldrich, A5268-10G, Missouri, USA) for determining TP and AP, respectively. The quantitative analysis of the protein used BIO-RAD DC protein Assay kit (Bio-Rad Laboratories AB, 500-0111, Oslo, Norway) using albumin as a standard for calibration.

## Results

### Effect of acidification on the growth of *E. huxleyi*

The growth curve of *E. huxleyi* determined by cell number and turbidity showed clear suppression by acidification with HCl under the aeration of ordinary air (Fig. 1a, b). The pH values of the medium in three cultures were maintained nearly constant with slight increases from 8.2 to 8.4 (8.2 for first 4 days), 7.7 to 7.9 (7.7 for first 4 days) and 7.2 to 7.3 (ca. 7.2 for first 4 days) during 7 days (Fig. 1c). The pH values for first 4 days were used to express culture conditions in the text. The specific growth rate (*μ*) decreased by acidification ca. 30 and 60 % at pH 7.7 and 7.2, respectively, in comparison with that at pH 8.2 (Fig. 1d). Cell growth at pH 7.2 was rapidly and strongly suppressed in a day, and then, cells were destroyed (Fig. 1a, b). The concentrations of total DIC and bicarbonate ions at pH 7.7 and 7.2 cultures were 75 and 90 % lower than that at pH 8.2 culture (Fig. 1e). As dissolved CO_2_ (dCO_2_) concentration in the medium is maintained as a constant according to the Henry’s law under bubbling of air, the suppression of growth at low pHs should be due to the combination of acidification effect and the decrease in HCO_3_^−^ concentrations equilibrated with air (Fig. 1e).

On the other hand, the growth of *E. huxleyi* was almost the same among different CO_2_ concentrations and pHs when acidification was performed by the bubbling of air containing elevated CO_2_ concentration such as 406, 816 and 1,192 ppm (acidification by CO_2_ enrichment) (Fig. 1f, g). During the culture for 7 d, the pH of the medium was maintained at 8.0–8.3, 7.6–7.9 and 7.5–7.7 by the bubbling of air containing 406, 816 and 1,192 ppm CO_2_, respectively (Fig. 1h). The specific growth rate (μ) was slightly higher ca. 15 and 25 % at 816 and 1,192 ppm CO_2_, respectively, in comparison with that at 406 ppm CO_2_ (Fig. 1i). Under such conditions, total DIC and bicarbonate concentrations were almost the same among the three different CO_2_ conditions resulting in different pHs (Fig. 1h) where dCO_2_ concentrations were increased according to the elevation of CO_2_ concentration (Fig. 1j).

### Effect of acidification on photosynthetic activity in *E. huxleyi*

**Fig. 2:**
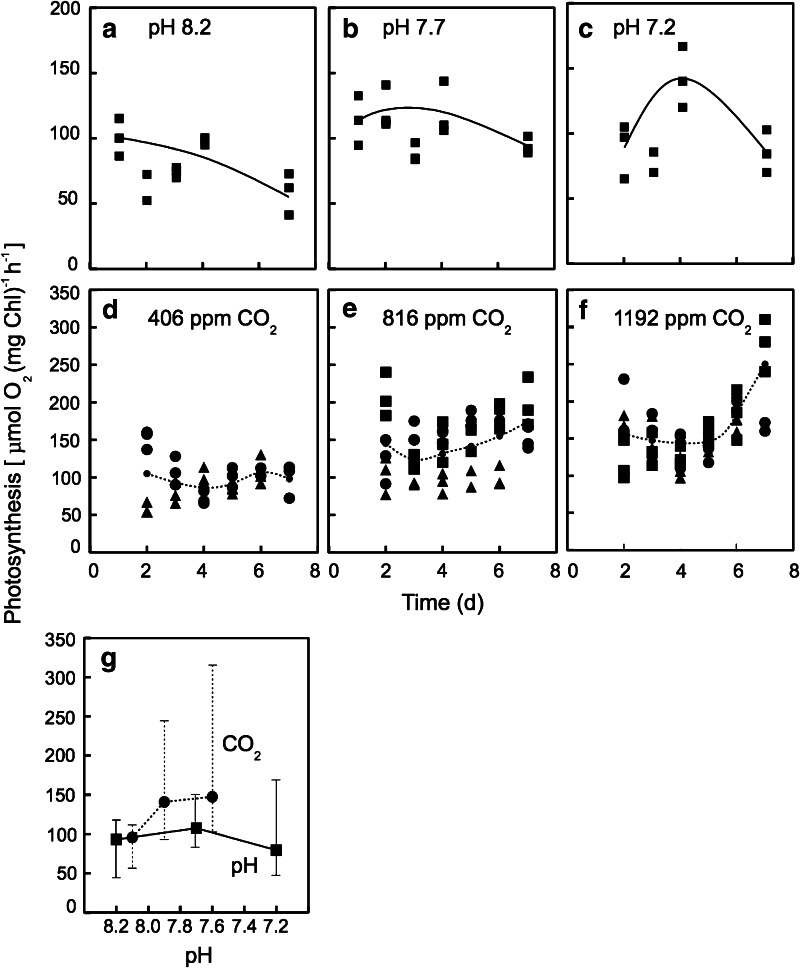
Effect of the acidification by HCl (**a**–**c**) and the ocean acidification conditions by elevating pCO_2_ (**d**–**f**) on the changes in photosynthetic O_2_ evolution activity of the coccolithophore *E. huxleyi*. Experimental conditions for acclimation (indicated in the figure) were same as shown in Fig. 1. The rate of photosynthetic O_2_ evolution was determined using a Clark-type O_2_ electrode at the light intensity of 270 μmol photons m^−2^ s^−1^ and 25 °C which are the optimum conditions. The values are average of three experiments (*n* = 3)

The photosynthetic O_2_ evolution activity was not affected when pH of the medium decreased (Fig. 2a–c, g), suggesting that photosynthetic machinery was hardly damaged by acidification with HCl. However, photosynthetic activity changed during the 7-day experiment at every pH tested. Although the reason is unclear yet, it maybe associated with the depletion of inorganic phosphate from the medium during growth, according to our previous study (Satoh et al. 2009). Photosynthetic O_2_ evolution activity was slightly higher at higher CO_2_ concentration when compared among the 406, 816 and 1,192 ppm CO_2_ experiments, where pH values were maintained at 7.9–8.3, 7.6–7.9 and 7.5–7.7 (Fig. 2d–f, g). The highest average value of photosynthetic O_2_ evolution was 150 μmol (mg Chl)^−1^ h^−1^ at pH 7.5–7.7, which was attained by the bubbling of air containing 1,192 ppm CO_2_ (Fig. 2g). These results show that the response of photosynthetic activity to pH change was almost the same, irrespective of the method of how pH was decreased, namely by adding HCl or bubbling air with elevated CO_2_.

**Fig. 3:**
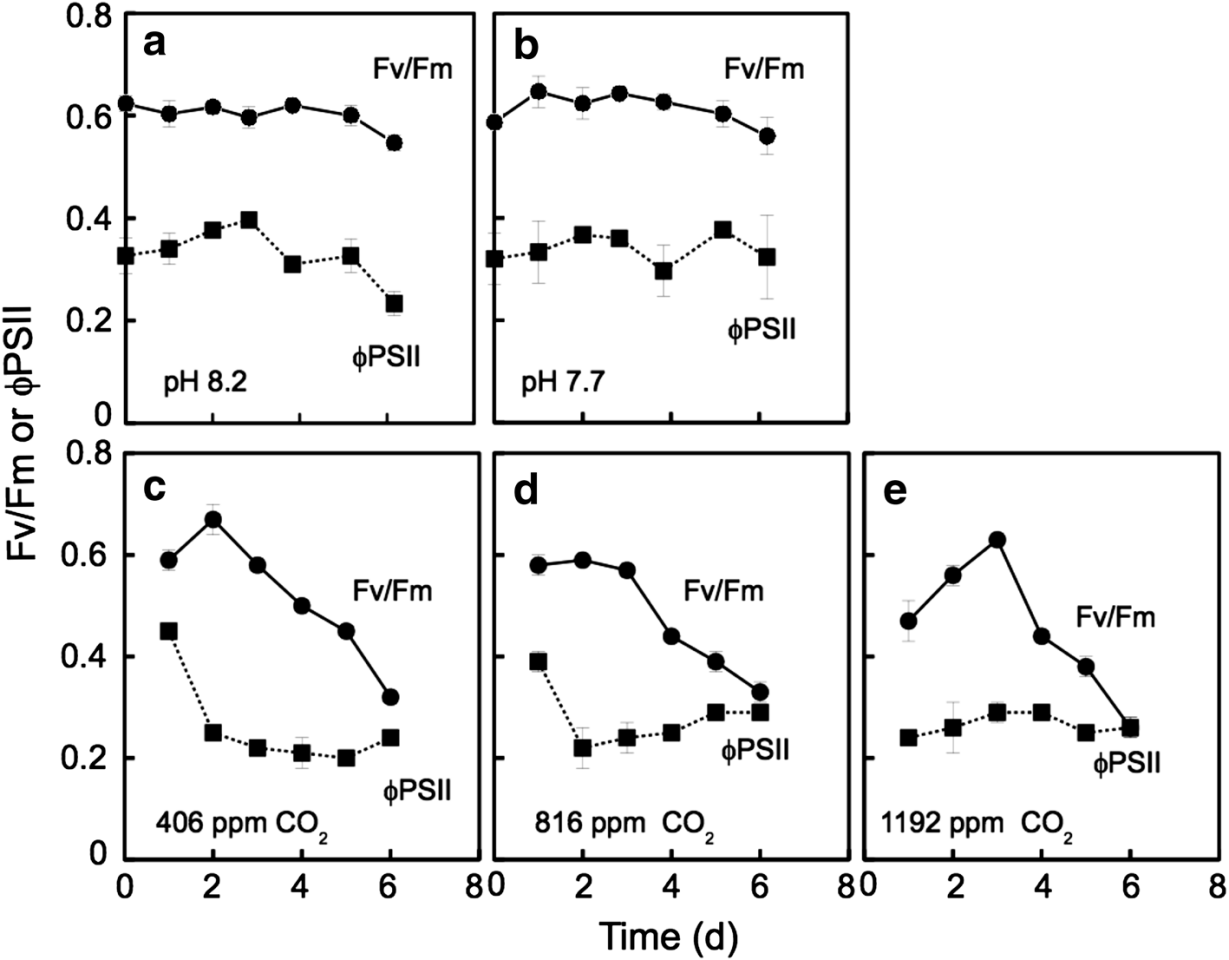
Effect of the acidification by HCl (**a**, **b**) and the ocean acidification conditions by elevating *p*CO_2_ (**c**–**e**) on the changes in the parameters of photosystem activity such as *F*_v_/*F*_m_ and ϕPSII during growth of the coccolithophore *E. huxleyi*. The chlorophyll fluorescence parameters were determined by Fluorcam, as described in “Materials and methods.” *Solid line* (*circles*), *F*_v_/*F*_m_; *dotted line* (*square*), ϕPSII. *Error bars* ±SD (*n* = 3)

The activities of the photosystems were determined by measuring *F*_v_/*F*_m_, which reflects the state of photosystem II (Demmig and Bjorkman 1987) and ϕPSII, which is an index of the electron transport activity of the whole photosystem (Genty et al. 1989). The results indicate that the photosystem parameters determined were not changed, namely almost the same, during the 6-day experiment between pH 7.7 and 8.2 (Fig. 3a, b). On the other hand, *F*_v_/*F*_m_ decreased similarly after 3 days under all tested CO_2_ conditions when pH was set by the bubbling of air containing various CO_2_ (Fig. 3c, e). Under the same conditions, ϕPSII was maintained almost constant for 6 days after rapidly decreasing during the first 2 days at 406 and 816 ppm CO_2_ conditions (Fig. 3c, d). There are no data on 0 day since the measurement of photosystem activities in the CO_2_ ventilation was begun after 1 day.

### Effect of acidification on coccolith production and calcification by *E. huxleyi*

**Fig. 4:**
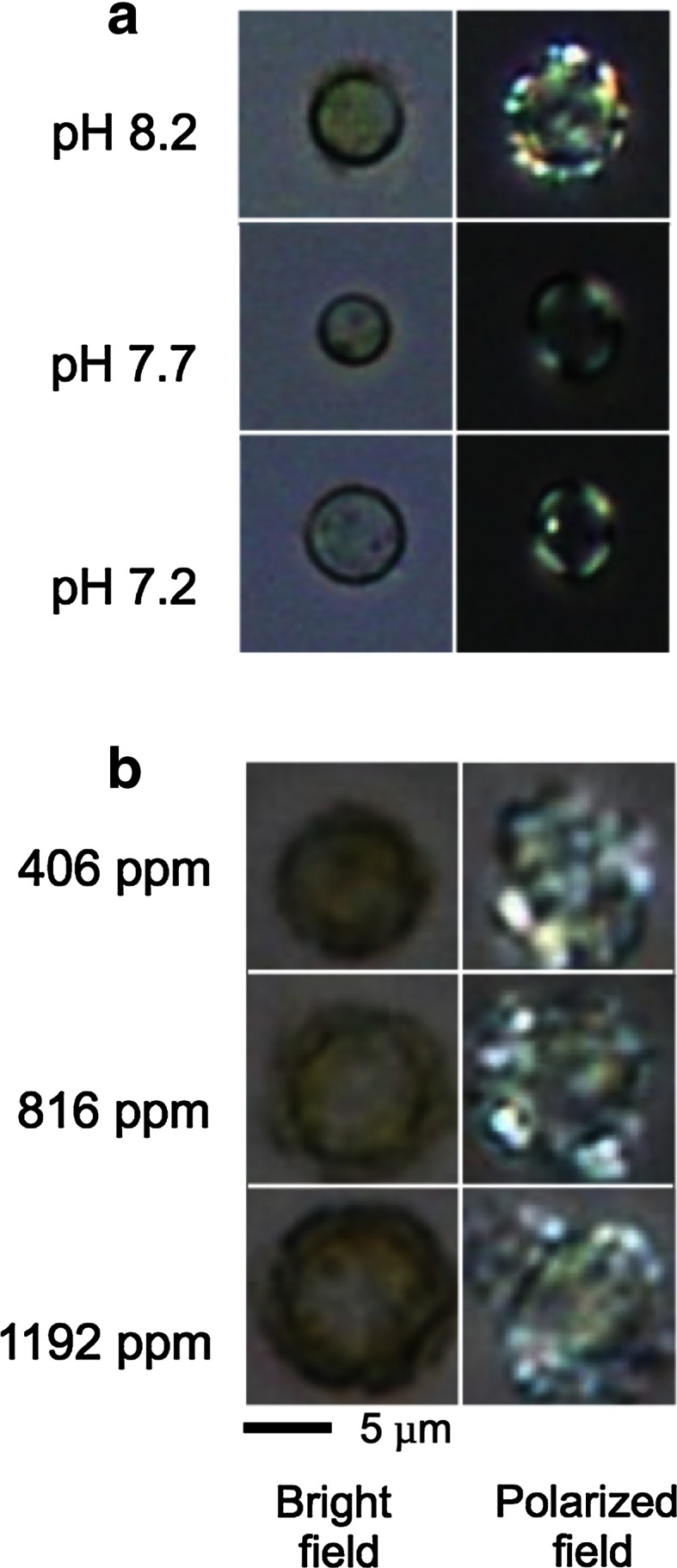
Effect of the acidification by HCl (**a**) and the ocean acidification conditions by elevating *p*CO_2_ (**b**) on the microscopic images for coccolith production and cell size of the coccolithophore *E. huxleyi*. The cells were grown for 12 days under each condition. Experimental conditions for acclimation (indicated in the figure) were same as shown in Fig. 1

Polarized light microscopic observations clearly showed that coccolith production was strongly suppressed when acidification was performed by HCl from 8.2 to pH 7.7 and 7.2 (Fig. 4a). In contrast, coccolith production was strongly stimulated and accompanied by an increase in cell size when pH was maintained at 8.0–8.3, 7.6–7.9 and 7.5–7.7 by the bubbling air containing various CO_2_ concentrations with 406, 816 and 1,192 ppm, respectively (Fig. 4b).

**Fig. 5:**
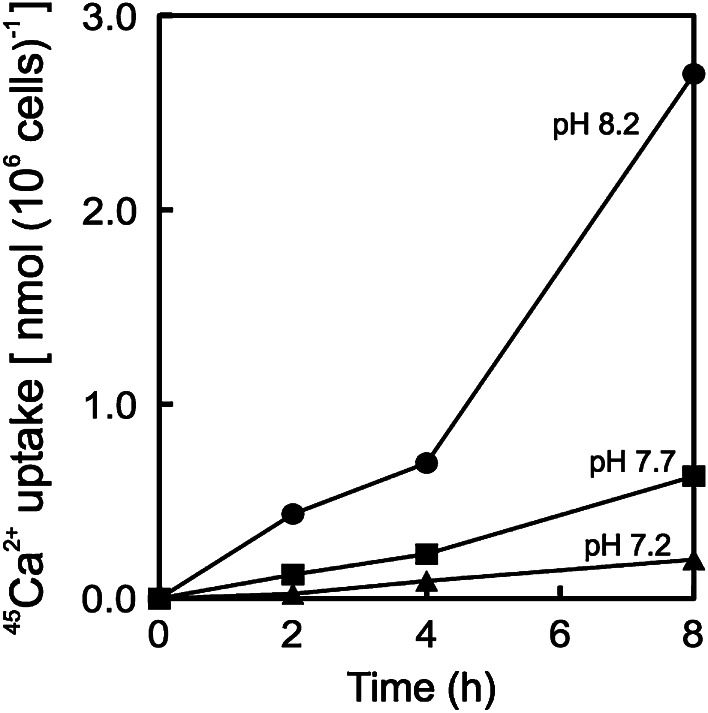
Effect of the acidification by HCl on ^45^Ca-uptake by the coccolithophore *E. huxleyi*. In order to stimulate coccolith production, cells grown for 12 days were transferred to the orthophosphate-free medium for the radiotracer experiments. The concentration and the specific radioactivity of ^45^Ca were 1 mM as CaCl_2_ and 20 MBq mmol^−1^, respectively. *Circles* pH 8.2; *squares* pH 7.7; *diamonds* pH 7.2

**Fig. 6:**
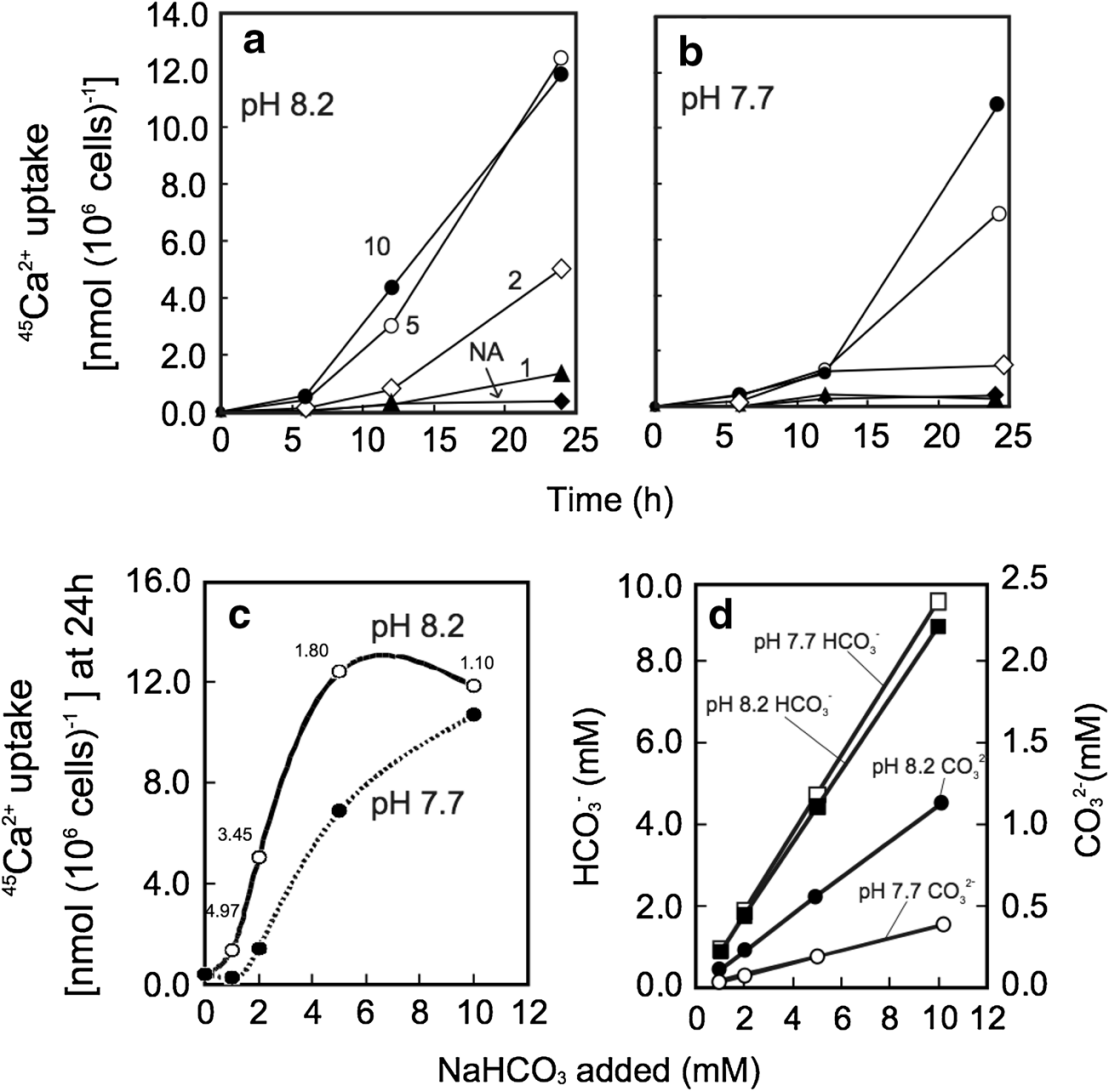
Effect of the acidification by HCl on ^45^Ca-uptake by the coccolithophore *E. huxleyi* under growth conditions. **a**, **b** Time course of ^45^Ca-uptake in the presence of various NaHCO_3_ concentrations at pH 8.2 and 7.7, respectively. The *numbers* beside of *lines* in **a** represent the final concentrations (mM) of NaHCO_3_ added to the medium: *NA* (not added, *filled diamond*), *1* (*filled triangle*), *2* (*open diamond*), *5* (*open circle*), *10* (*filled circle*). **c** Relationship between ^45^Ca-uptake activity during 24 h and the final concentrations of NaHCO_3_ added to the medium. The *numbers* beside of pH 8.2 *line* indicate the ratios of values at pH 8.2–7.7. **d** HCO_3_^−^ concentrations in the medium containing various concentrations (final) of NaHCO_3_ at pH 8.2 and 7.7. The equilibration of inorganic carbons was calculated by CO_2_ SYS

*E. huxleyi* needs to incorporate and accumulate calcium and bicarbonate ion as substrates for intracellular coccolith production into the coccolith vesicles within the coccolithophore cells. The rate of ^45^Ca-incorporation activity was strongly suppressed to 22 and 7 % at 7.7 and 7.2, respectively, in comparison with that of pH 8.2 when pH values were set by acidification with HCl under continuous bubbling of ordinary air (Fig. 5). When the concentration of CO_2_ dissolved in the solution is equilibrated with atmospheric air, bicarbonate concentration is calculated to be almost the same between pHs 8.2 and 7.7, but carbonate concentration is much higher at pH 8.2 than 7.7 (Fig. 6d). These data clearly show that ^45^Ca-incorporation into cells was greatly diminished by acidification with HCl, although the concentration of bicarbonate, the substrate to be absorbed by cells for intracellular calcification (Sekino and Shiraiwa 1994), was the same at both pHs.

On the other hand, ^45^Ca-incorporation activity was stimulated by the addition of DIC (NaHCO_3_) regardless of concentration (Fig. 6a, b). Under such conditions, the ^45^Ca-uptake activity was largely stimulated and saturated with 5 mM NaHCO_3_ at pH 8.2, but not completely even with 10 mM at pH 7.7 while the extent of suppression by acidification was the largest at 1–2 mM DIC (Fig. 6c). These results indicate that the suppression of ^45^Ca-uptake by acidification with HCl can be recovered by the addition of NaHCO_3_, namely by the increase in bicarbonate concentration.

### Effect of acidification on the production of coccolith polysaccharides by *E. huxleyi*

**Fig. 7:**
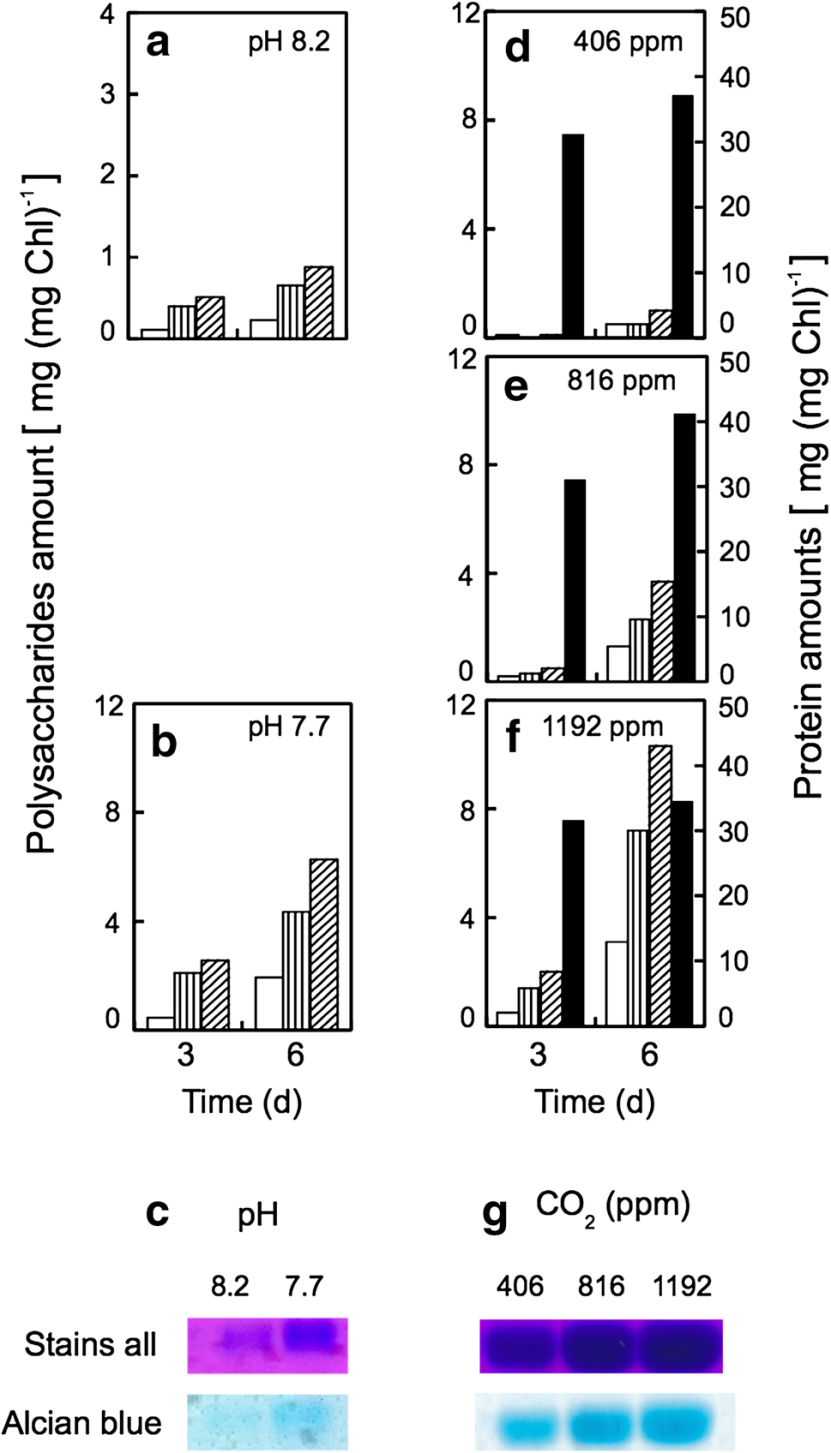
Effect of the acidification by HCl (**a**, **b**) and the ocean acidification conditions by elevating pCO_2_ (**c**–**e**) on the production of polysaccharide and proteins by the coccolithophore *E. huxleyi* during 3 and 6 days under growth conditions. **a**, **b** At pH 8.2 and 7.7, respectively. **d**–**f** Under the bubbling of 406, 816 and 1,192 ppm CO_2_ in air of which pH attained were 8.0–8.3, 7.6–7.9 and 7.5–7.7, respectively, as indicated in the figure. Before experiments, cells had been grown at pH 8.2. *White column* acid polysaccharides (AP) determined by the carbazole-sulfuric acid method; *vertical stripe column* neutral polysaccharides (NP) calculated by the equation of [TP] − [AP]; *hatched column* total polysaccharides (TP) determined by the phenol–sulfuric acid method; *black bar* protein contents determined by the protein assay kit (Bio-Rad Laboratories AB). **c**, **g** Effect of acidification by HCl (**c**) and acidification by CO_2_ enrichment (**g**), respectively, on SDS-PAGE image for the production of neutral and acid polysaccharides by the coccolithophore *E. huxleyi* grown for 6 days. The amount of cell used for analysis was corresponded to 5 μg Chl. Total and acid polysaccharide bands were visualized by “Stains-all” and “Alcian blue,” respectively

Acidification by CO_2_ enrichment stimulated the production of cellular contents of photosynthetic storage products such as neutral (NP) and acid (AP) polysaccharides, which are located in the cytoplasm and coccoliths, respectively, at pH 7.7 in comparison with pH 8.2 (Fig. 7a, b). On the other hand, the content of those polysaccharides was remarkably increased when acidification was attained by CO_2_ enrichment (Fig. 7d–f). The quantitative analytical data of NP and AP were also confirmed by SDS-PAGE images (Fig. 7c, g). The ratio of the amount of AP/NP was not affected by acidification with HCl (Fig. 7a, b), but NP production was more stimulated by acidification with CO_2_ enrichment (Fig. 7d–f).

## Discussion

According to the IPCC scenario, oceanic pH is estimated to decrease 0.5 U, namely to pH 7.7, by 2100 (IPCC 2007). In addition to the effects of atmospheric CO_2_ elevation, acidification also can be seen at shallow coastal sites of volcanic CO_2_ vents. Along gradients of normal pH (8.1–8.2) to lowered pH (7.8–7.9, lowest 7.4–7.5), typical rocky shore communities with abundant calcareous organisms shifted to communities lacking scleractinian corals with significant reductions in sea urchin and coralline algal abundance (Hall-Spencer et al. 2008). If it happens in the surface ocean, coccolithophores will also be damaged and such damage of the primary producers in the ocean will change the composition of the global phytoplankton community and ecosystems. There are various views on the effect that ocean acidification has on calcification of the coccolithophore *E. huxleyi*. Algal growth was reported to be suppressed by acidification in coccolithophores, e.g., the decrease in the specific growth rate of coccolithophores at pH values below 8.0 (Swift and Taylor 1966). Iglesias-Rodriguez et al. (2008) reported that promotion of the calcification would happen by increase of the CO_2_. In contrast, Riebesell et al. (2000) described that the formation of the coccoliths will be inhibited by acidification.

In this study, we intended to compare the difference of acidification effect between acidification by acid supply and the bubbling of elevated concentrations of CO_2_ in order to observe how coccolithophores respond potentially to acidification. The experimental conditions set in this study were not exactly the same as those expected in ocean acidification since seawater contained buffers to induce change in alkalinity. Cell density was also very high, and the rate of bubbling was not strong enough to get complete equilibration of inorganic carbons. Therefore, while the data we obtained are not directly applicable to the determination of the effect of ocean acidification on coccolithophores in the ocean, the data are still useful to predict how coccolithophores will respond to acidification physiologically. For this purpose, we analyzed the whole effect of acidification on cell growth, photosynthetic O_2_ evolution, photosystem’s activity, Ca-uptake, the productivity of polysaccharides of AP and NP and coccolith production in the most abundant, bloom-forming coccolithophore, *E. huxleyi*.

When pH was simply decreased to 7.7 by acidification with HCl, the specific growth rate of *E. huxleyi* was diminished 31.2 % lower than that at pH 8.2 and they rapidly died within 1 day at pH 7.2 (Fig. 1a–d). In contrast, the acidification by CO_2_ enrichment by bubbling of 816 (lowest pH 7.6) and 1,192 ppm (lowest pH 7.5) slightly promoted algal growth (Fig. 1f–i). Those results indicate that *E. huxleyi* responds differently to acidification depending on whether it is accompanied by CO_2_ enrichment or not. The results also show that the diminution of algal growth by acidification with HCl can be overcome by an increase in CO_2_ supply.

Acidification shifts DIC equilibrium toward CO_2_, and therefore, the concentration of total DIC becomes low when pH is decreased in an open system (Fig. 1e). Interestingly, bicarbonate concentration calculated was almost similar at pH 8.2 and 7.7 at constant dissolved CO_2_ concentration under bubbling of air (Fig. 6d). The radiotracer experiment on ^45^Ca-uptake by *E. huxleyi* cells was performed to analyze the effect of acidification by HCl under bubbling of air with ca. 400 ppm. The results of the experiments clearly showed that ^45^Ca-uptake was strongly suppressed by acidification with HCl (Fig. 5). However, ^45^Ca-uptake was saturated with 5 mM DIC at pH 8.2, but not enough with 10 mM at pH 7.7 (Fig. 6c), indicating that high bicarbonate concentration is required for calcification. This result agrees with evidence showing that only bicarbonate, not CO_2_, is the substrate for intracellular calcification on *E. huxleyi* (Sekino and Shiraiwa 1994). Although the influence of acidification on calcification of *E. huxleyi* has been reported (Zondervan et al. 2001; Riebesell et al. 2000; Langer et al. 2006; Iglesias-Rodriguez et al. 2008), the mechanism how acidification changes physiological status of coccolithophores has not been studied in detail. Therefore, the present result gives important information to elucidate how acidification by acid and by CO_2_ enrichment will be different.

**Fig. 8:**
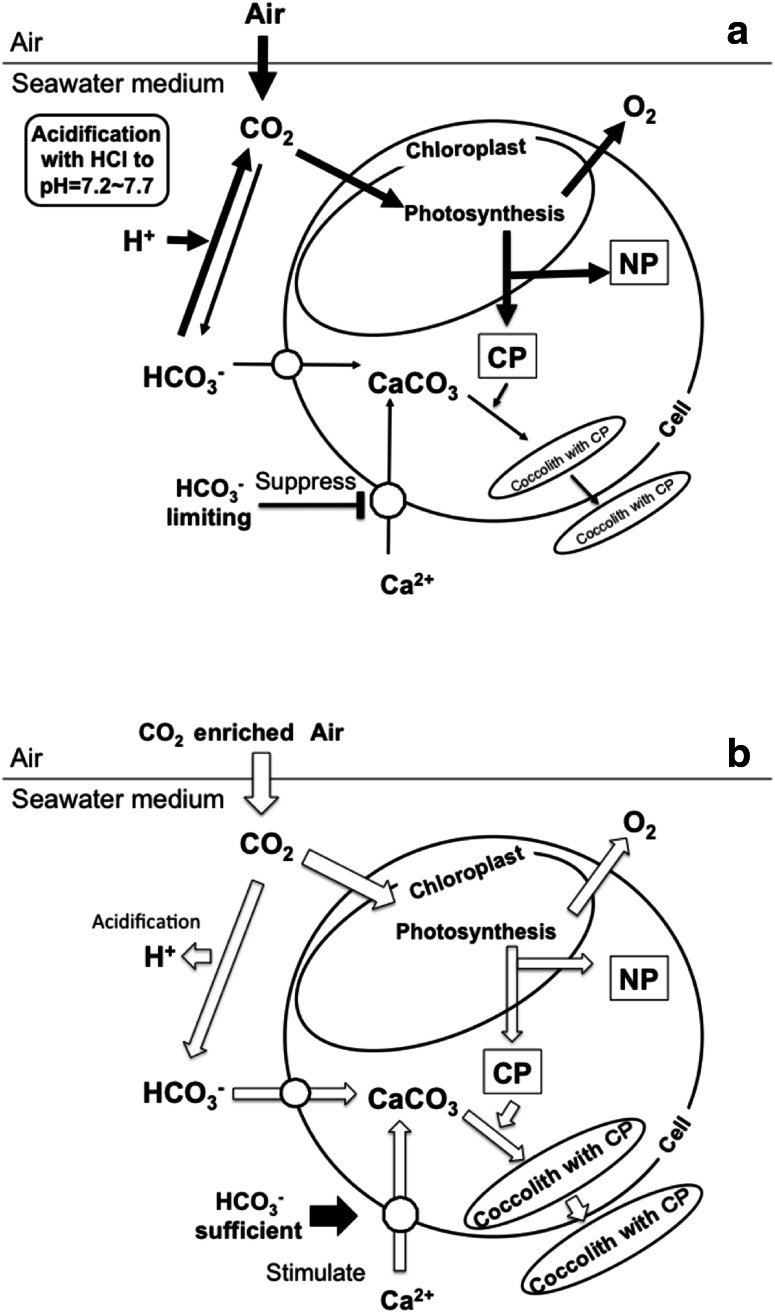
Schematic models showing the effect of acidification by HCl (**a**) and by bubbling of air with elevated CO_2_ (**b**) on CO_2_ equilibration, inorganic carbon utilization for photosynthesis, Ca-uptake and coccolith production by the coccolithophore *E. huxleyi*. The width of arrows represents the amount of compounds how much flow along the *arrow*. **a** Acidification by HCl changes in the equilibration between dissolved CO_2_ and bicarbonate toward CO_2_ production to decrease bicarbonate concentration. Dissolved CO_2_ concentration equilibrated with air bubbled was same among three pH conditions. The present study proved that the decrease in HCO_3_^−^ concentration suppressed coccolith production which is due to diminishing Ca^2+^-uptake by cells. Photosynthetic production of storage (NP) and coccolith polysaccharides (CP) was stimulated by acidification. **b** Acidification by CO_2_ enrichment increases dissolved CO_2_ concentration and bicarbonate production by increasing inorganic carbon substrates. The resulted increase in CO_2_ and bicarbonate which are substrate for photosynthetic CO_2_ fixation and intracellular calcification, respectively (Sekino and Shiraiwa 1994), stimulated both reactions. High concentration of bicarbonate also stimulated Ca-uptake. As a result, all those processes stimulated photosynthetic CO_2_ fixation and coccolith production

In unicellular green alga *Mesotaenium caldariorum*, the high rate of ATP-dependent Ca^2+^-uptake and direct Ca^2+^-transport/H^+^-antiport activities was found to be necessary for Ca^2+^ uptake (Berkelman and Lagarias 1990). Ca^2+^-permeable channels in the plasma membrane were suggested more likely to function for Ca^2+^ entry into calcifying coccolithophore cells (Brownlee and Taylor 2003). Ca^2+^ accumulation into the Golgi of eukaryotic cells occurs by H^+^/Ca^2+^ exchange driven by the inside acidic H^+^ electrochemical gradient across the Golgi membrane, which in turn is generated by V-type ATPase in eukaryotic cells (Harvey 1992). These previous reports show that acidification outside of membrane may disturb Ca^2+^ uptake through the Ca^2+^/H^+^ channel. The results support our conclusion that the suppression of Ca^2+^-uptake, calcification and coccolith production by *E. huxleyi* is due to the suppression of Ca^2+^-entry into cells by acidification of the medium (solid line in Fig. 8a). In addition, as the calcite saturation state is <1 in the low pH cultures, the coccoliths may also be dissolved even though coccoliths were produced and transported to the cell surface.

Acidification by CO_2_ enrichment promoted photosynthetic O_2_ evolution (Fig. 2), the morphological change in increase of cell volume, coccolith production (Fig. 4), Ca^2+^-uptake into cells (Fig. 6), and the production of acid (AP) and neutral polysaccharides (NP) (Fig. 7). On the other hand, acidification by both HCl and CO_2_ enrichment did not affect the activity of photosystem activities directly (Fig. 3).

The state of photosystem II determined by *F*_v_/*F*_m_ and the electron transport activity of the whole photosystem (ϕPSII) of acidification were hardly changed by acidification during growth, irrespective of pH of the medium (Fig. 3a, b). In contrast, the *F*_v_/*F*_m_ values decreased under ocean acidification conditions where air with elevated concentration of CO_2_ was bubbled (Fig. 3c, e). The reason for the difference is unclear yet. These data are different from a previous report in which damage of photosystem II (PSII), namely decrease in *F*_v_/*F*_m_, by acidification in the thylakoid membrane of the green algae *Scenedesmus obliquus* (Heinze and Dau 1996). The possible explanation on the phenomena is that excess CO_2_ concentration in the medium induces high CO_2_ input into the chloroplast stroma and results in rapid acidification by the conversion of CO_2_ to bicarbonate plus H^+^ by chloroplast carbonic anhydrases. Those reactions affect PSII directly and induced a decay of the *F*_v_/*F*_m_ value.

Acid (AP), same as coccolith polysaccharide (CP), and neutral polysaccharides (NP) determined as parameters of coccolith and storage polysaccharides (Kayano and Shiraiwa 2009), respectively, are known as coccolith polysaccharides containing uronic acids and storage polysaccharides composed of β-1-3(1–6)-glucan, respectively (Mann 2001; Kayano and Shiraiwa 2009). The amount of AP and NP production was stimulated by acidification, but the AP/NP ratio was not affected (Fig. 7). These phenomena may be due to an increase of CO_2_ supply into the cells and consequently the stimulation of the production of acid polysaccharides. Such active AP production also may stimulate Ca^2+^-uptake by demand of Ca^2+^ to produce CaCO_3_ crystals for coccoliths. Both cell size and coccolith production were affected by acidification with CO_2_ concentration (Fig. 4). Cell enlargement was also observed when coccolith production was strongly stimulated at low temperature (Sorrosa et al. 2005). As swelling of the cells were observed when cell growth was greatly suppressed by nutrient-deficiency or cell damage (Satoh et al. 2009), cell enlargement by acidification with HCl to pH 7.2 might be due to cell damage. Satoh et al. (2009) and Kayano and Shiraiwa (2009) also reported that both coccolith and coccolith polysaccharide production were stimulated by phosphate deficiency from the medium, although the reason why cell size was enlarged by phosphate deprivation is still unclear.

Very recently, Bach et al. (2013) reported the results on analysis of impact of CO_**2**_ and pH on the mechanism of photosynthesis and calcification in *E. huxleyi* and concluded that *E. huxleyi* is sensitive to low CO_**2**_ and low bicarbonate as well as low pH beyond a limited tolerance range, but much less sensitive to elevated CO_**2**_ and bicarbonate. These results nicely fit to our present results although the parameters determined experimentally in both studies were different. The experiments by Bach et al. (2013) were performed by following carbon chemistry exactly, and therefore, their results can be extrapolated to the real ocean to simulate how *E. huxleyi* will be affected by ocean acidification. The present study clearly proved the mechanism behind how and why calcification, namely coccoliths production, is stimulated at elevated CO2 conditions and inhibited under acidification. Therefore, the combination of both papers is useful to understand how and why ocean acidification by increasing atmospheric CO_**2**_ will affect the physiology of the coccolithophore *E. huxleyi*.

In conclusion, the schematic model of the influence of acidification by acid (solid arrow) and by CO_2_ enrichment (open arrow) is shown in Fig. 8. The suppression of coccolith formation by acidification is shown to be due to the reduction of calcium uptake through the plasma membrane in *E. huxleyi*. On the other hand, photosynthetic machinery in the chloroplast was not affected by such acidification of the medium. This study proved that *E. huxleyi* cells have high potential of compensation to avoid damage of cells against acidification when acidification is caused by CO_2_ enrichment. This suggests that physiological activities of *E. huxleyi* cells will not be seriously damaged by ocean acidification at least up to 1,200 ppm CO_2_ in the atmosphere. However, as reported by Hoppe et al. (2011), there still is possibility that some ecophysiological difference and/or variation of *E. huxleyi* strains living in some specific habitats may induce some different response to ocean acidification.
